# Angioscopy-assisted thoracic endovascular aortic repair for chronic type B aortic dissection: Optimizing stent graft coverage and left subclavian artery coil embolization

**DOI:** 10.1016/j.jvscit.2025.102058

**Published:** 2025-11-21

**Authors:** Yukihisa Ogawa, Hiroyuki Nishi, Hidekazu Furuya, Ryuichi Tamimoto, Satoru Takahashi, Shunsuke Kamei

**Affiliations:** a Department of Radiology, Tokai University Hachioji Hospital, Hachioji, Japan; b Department of Cardiovascular Surgery, Tokai University Hachioji Hospital, Hachioji, Japan; c Department of Cardiology, Osaka Gyomeikan Hospital, Osaka, Japan

**Keywords:** Aortic remodeling, Coil embolization, Left subclavian artery, Nonobstructive general angioscopy, TEVAR

## Abstract

We report the case of a 69-year-old man with chronic type B aortic dissection and progressive aneurysmal dilatation. Zone 2 thoracic endovascular aortic repair was performed with carotid-subclavian bypass. Nonobstructive general angioscopy identified additional tiny entry tears in the descending aorta, guiding adequate stent graft coverage. Subsequent left subclavian artery coil embolization under balloon occlusion revealed satisfactory packing on fluoroscopy, but angioscopy revealed residual flow through coil gaps, prompting additional coil placement. Blood flow cessation was confirmed by angioscopy. Completion angiography revealed no antegrade false lumen flow or endoleaks. Follow-up computed tomography at 3 months showed favorable aortic remodeling with complete thoracic false lumen thrombosis.

Type B aortic dissection (TBAD) often extends immediately distal to the left subclavian artery (LSA), necessitating zone 2 thoracic endovascular aortic repair (TEVAR) with intentional coverage of the LSA.

Nonobstructive general angioscopy (NOGA) has recently been reported to be a useful adjunct in TEVAR for TBAD.^1^ This is an intravascular endoscopic technique that enables direct, real-time visualization of minor entry tears and intimal surface irregularities that are difficult to identify on computed tomography (CT) under continuous saline flushing without interrupting blood flow. It facilitates appropriate stent graft coverage and promotes favorable aortic remodeling.^1^^,^^2^

Contemporary guidelines recommend routine LSA embolization with revascularization with zone 2 coverage to prevent endoleaks and spinal cord ischemia.^3^^,^^4^

Robust evidence specifically demonstrating undiagnosed endoleaks after LSA embolization is limited. Therefore, we have focused on the current case to highlight the potential of NOGA as a practical adjunct providing direct visualization beyond angiography.

Here, we report a case of zone 2 TEVAR for chronic TBAD with false lumen expansion, in which NOGA was instrumental in identifying minor entry tears for stent graft coverage and in determining the endpoint of LSA coil embolization.

## Case report

A 69-year-old male with a history of chronic TBAD diagnosed 7.5 years earlier, presented with aneurysmal dilatation (zone 3) that had progressed from 35 mm at onset to 53 mm on follow-up CT.

His medical history included right ureteral cancer, which had been treated with nephroureterectomy and subsequent chemotherapy for lymph node metastases.

**Fig 1:**
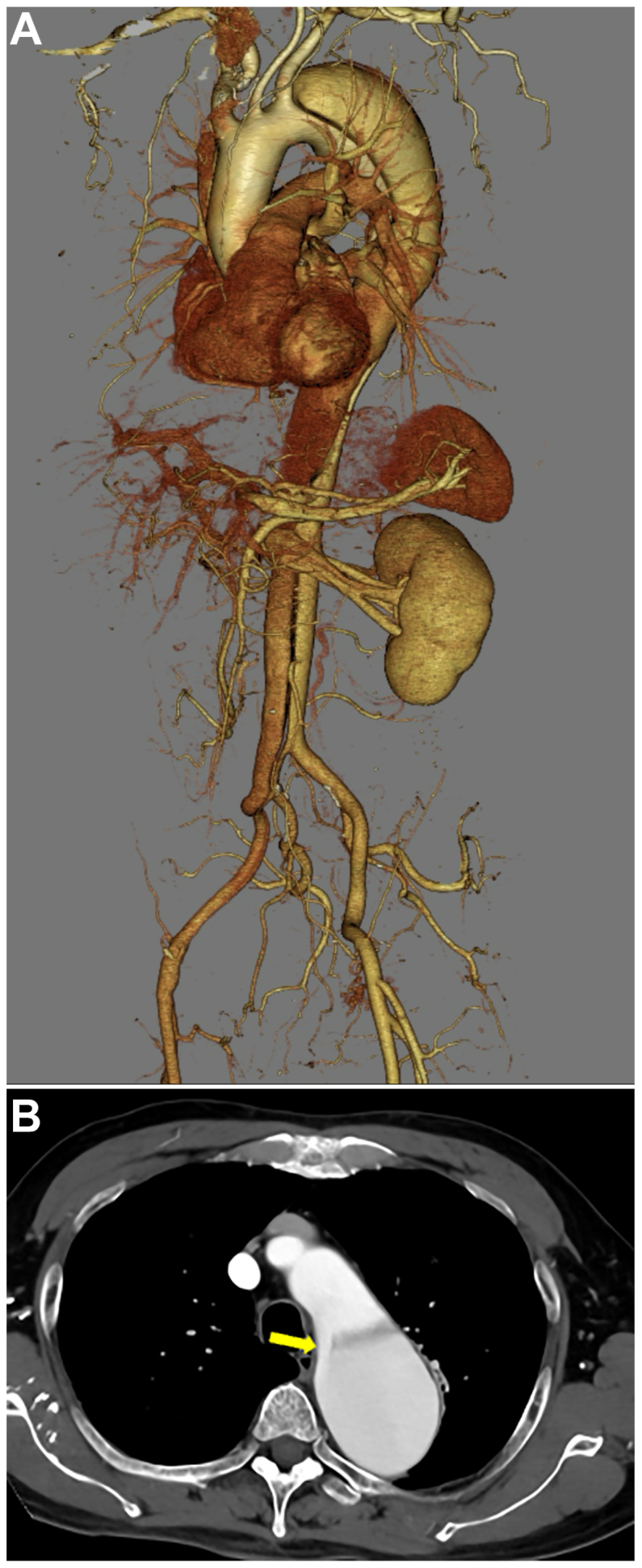
Volume rendering of computed tomography (CT) angiography demonstrates a chronic type B aortic dissection (TBAD) extending from just distal to the left subclavian artery (LSA) to the right external iliac artery **(A)**. Axial CT image shows the primary entry tear (*arrow*) in the distal aortic arch **(B)**.

The dissection extended from just distal to the LSA down to the right external iliac artery (Fig 1, *A*). The primary entry tear was located in the distal arch (Fig 1, *B*), and several re-entry tears were identified from the abdominal aorta to the right iliac artery. No entry tears were observed in the descending aorta on CT. Due to the enlarged false lumen in the thoracic aorta, we planned zone 2 TEVAR to close the primary entry tear.

Under general anesthesia, a left carotid-subclavian bypass was performed in the same operative session prior to TEVAR. We subsequently evaluated the aortic intima using a NOGA system comprising a VISIBLE fiber (FiberTECH Co, Ltd), a Fiber Imaging System FT-203F (FiberTECH Co, Ltd), and a console (Intertec Medicals Co, Ltd). The detailed specifications and methodology of this system have been described previously.^5^ The tips of the fiber catheter, 4F probing catheter, and 6F guiding catheter were set at the same position in the distal arch and then gradually pulled back to the abdominal aorta at the level of the celiac trunk.

**Fig 2:**
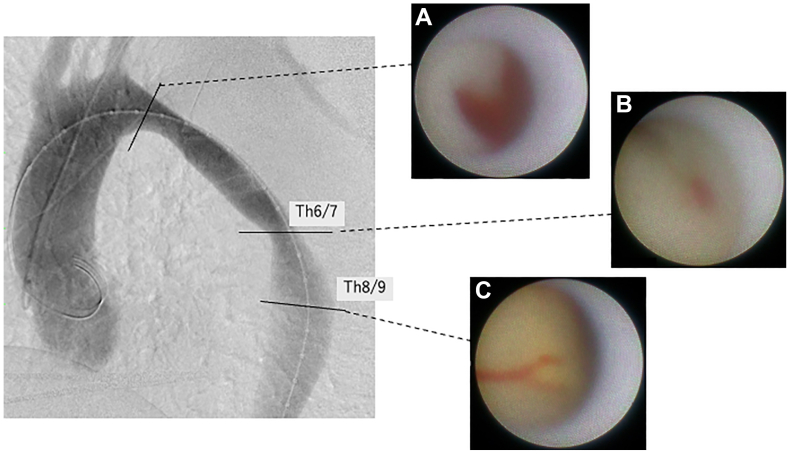
Aortography (left oblique sagittal view) and corresponding angioscopic images. Nonobstructive general angioscopy (NOGA) reveals the primary entry tear in the distal arch **(A)**, and two tiny entry tears located at the Th6/7, and Th8/9 levels of the descending aorta **(B and C)**.

Angioscopy revealed a primary entry tear in the distal arch and two tiny entry tears located at the Th6/7 and Th8/9 levels of the descending aorta (Fig 2). The proximal landing zone diameter was 31 mm, and the distal landing zone flap length was 27 mm. To cover these entries, two overlapping stent grafts (Gore TAG conformable; W. L. Gore & Associates, Inc; 34 mm × 15 cm and 28 mm × 10 cm) were deployed to ensure minimal necessary coverage.

After stent graft deployment, a 9-mm balloon catheter (Selecon MP catheter, Terumo) was introduced via a 5F sheath into the left radial artery, and balloon occlusion of the LSA was performed.

**Fig 3:**
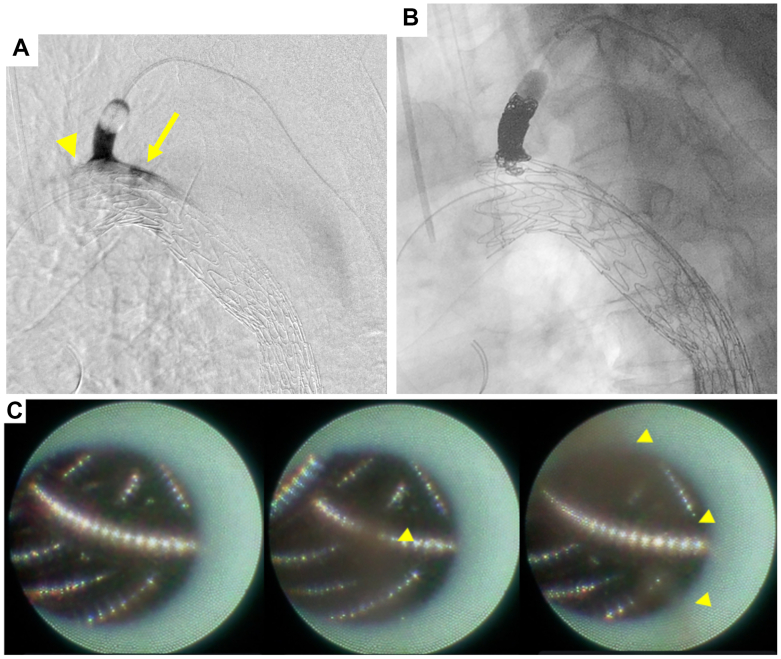
Left subclavian artery (LSA) arteriography under balloon occlusion demonstrated visualization of the false lumen, suggesting a type II endoleak (*arrow*). In addition, a leak was observed at the proximal perigraft (*arrowhead*), indicating the presence of a type Ia endoleak. Fluoroscopy suggests satisfactory coil packing in the LSA **(B)**, but nonobstructive general angioscopy (NOGA) reveals residual blood flow (*arrowhead*) through small gaps between coils **(C)** (See Supplementary Video 1, online only).

Under balloon occlusion, LSA angiography revealed type II endoleak and contrast leakage through the proximal sealing perigraft space, suggesting a type Ia endoleak (Fig 3, *A*).

**Fig 4:**
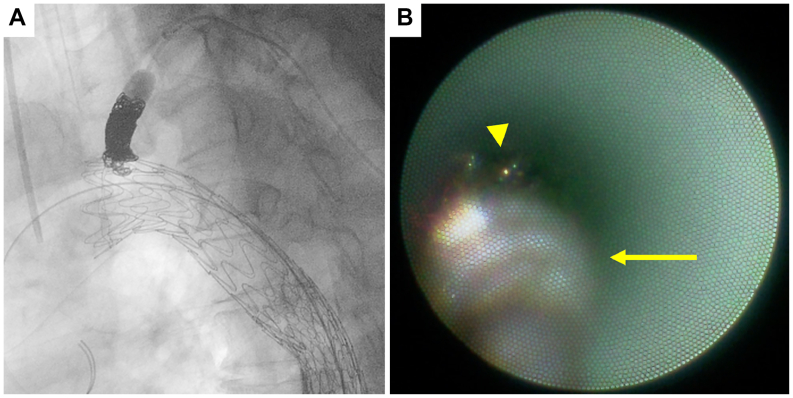
Deployment of an additional coil in the left subclavian artery (LSA) **(A)**, with cessation of blood flow confirmed by angioscopy **(B)**. (*arrowhead*, previous coils; *arrow*, additional coil) (see Supplementary Video 2, online only).

Using a 2.7F Coiling Support EX (MEDICO’S HIRATA Inc), 14 mm × 60 cm and 8 mm × 60 cm coils (POD; MEDICO’S HIRATA Inc.) were deployed within the LSA. Fluoroscopy revealed satisfactory coil packing (Fig 3, *B*). The fiber catheter was introduced through a balloon catheter to observe the coil embolization. Subsequent NOGA revealed residual blood flow through small gaps between the coils, indicating persistent antegrade flow (Fig 3, *C*; Supplementary Video 1, online only). An additional 6 mm × 50 cm coil (POD) was thus deployed to achieve complete embolization (Fig 4, *A*).

**Fig 5:**
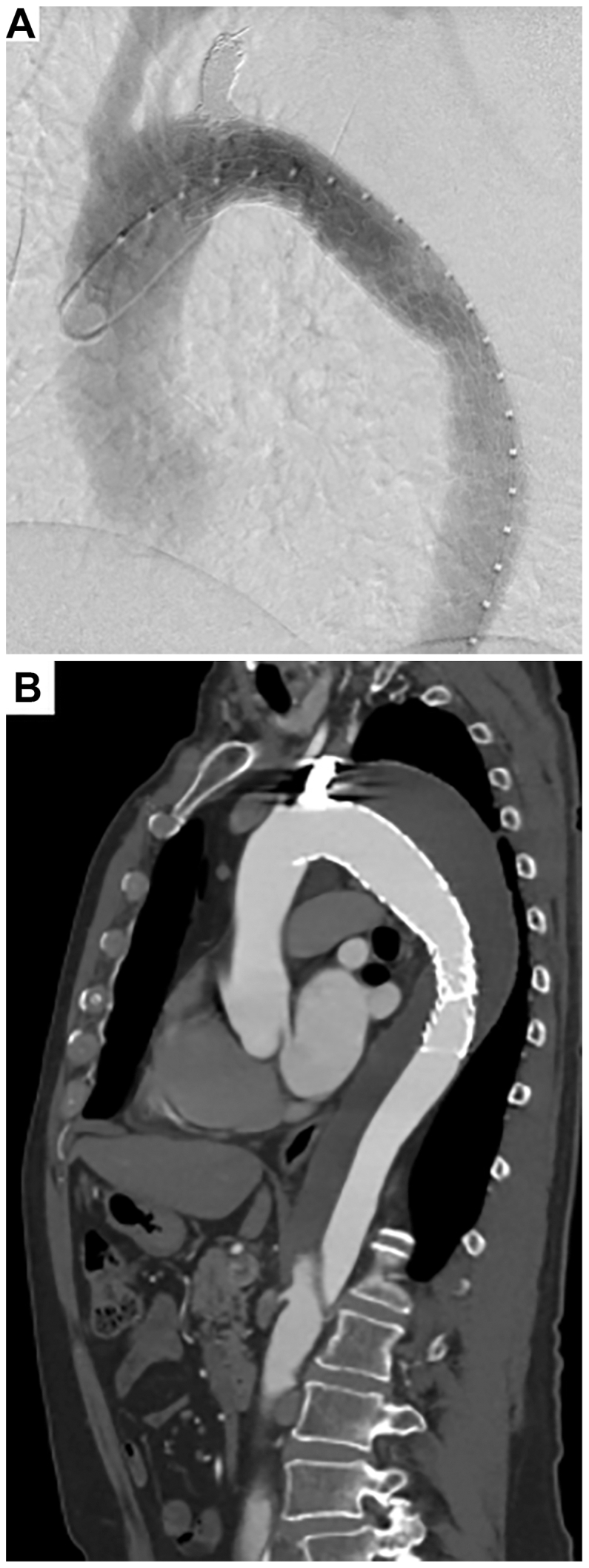
Completion angiography revealed no antegrade false lumen flow or endoleaks **(A)**. Follow-up computed tomography (CT) angiography at 3 months demonstrated favorable aortic remodeling with complete false lumen thrombosis in the thoracic aorta **(B)**.

After placement of the additional coil, NOGA confirmed cessation of blood flow (Fig 4, *B*; Supplementary Video 2, online only). Completion angiography revealed no antegrade false lumen flow or endoleaks (zone 3 to 4; Fig 5, *A*), and the procedure was completed without complications.

The postoperative course was uneventful, and the patient was discharged on postoperative day 7. Follow-up CT at 3 months revealed favorable aortic remodeling with complete false lumen thrombosis in the thoracic aorta (Fig 5, *B*). The patient expressed consent to the publication of this case report.

## Discussion

This case highlights the utility of NOGA in optimizing stent graft coverage and determining the best endpoint for LSA coil embolization.

Recent studies have emphasized that direct visualization of minor entries or intimal injuries that are not evident on CT allows adequate stent graft coverage, promoting favorable aortic remodeling.^1^^,^^4^^,^^6^

Angioscopic assessment also proved valuable in defining the endpoint of left subclavian coil embolization. Direct visualization of residual flow through the coils enabled additional coil placement, ultimately achieving complete embolization. To the best of our knowledge, this is the first report describing angioscopic assessment following LSA coil embolization.

Compared with intravascular ultrasound, which could serve as an alternative modality, the NOGA system is approximately 2.5 times more expensive. However, NOGA provides distinct advantages, enabling direct and dynamic visualization of the vessel lumen and allowing assessment of minor entries or residual blood flow that cannot be evaluated by intravascular ultrasound.

Simple coverage of the LSA without embolization using a stent graft reportedly increases the risks of type II and type Ia endoleaks, highlighting the importance of adjunctive LSA embolization.^7^, ^8^, ^9^ However, incomplete coil embolization of the LSA carries the risk of distal coil migration due to residual antegrade flow, making it critical to dense and complete embolization.^9^

Although Amplatzer vascular plugs (AVPs; Abbott) are increasingly used for LSA embolization, concerns remain regarding incomplete occlusion or potential recanalization. The coil-in-plug technique has recently been introduced as a method to enhance the embolic effect.^10^, ^11^, ^12^ However, AVP devices require a sheath size of ≥6F depending on vessel diameter, increasing the risk of access site injury. Although coils allow embolization in the perigraft space to prevent type Ia endoleaks, this cannot be achieved using AVP.

The ideal coil embolization strategy is to achieve tight packing within a minimally necessary segment of the vessel. Intrasaccular coil packing reportedly requires a volume embolization ratio of ≥24% for effective embolization,^13^ which is widely applied in clinical practice. However, no clear consensus exists regarding the optimal volume embolization ratio for parent artery embolization, and no criteria have been established for the appropriate length or density of coil placement in the LSA.

NOGA, which can be introduced through a 5F balloon catheter, is minimally invasive and readily applicable in such procedures. In the present case, although fluoroscopy revealed satisfactory coil packing, angioscopic observation revealed residual blood flow between coil gaps, prompting additional coil placement. This finding highlights the utility of NOGA in identifying incomplete embolizations that may not be apparent on angiography alone, thereby contributing to preventing postoperative endoleaks. NOGA may also be useful for assessing whether coils provide more complete embolization than AVP.

Given its small caliber and ease of use, NOGA may be actively employed as a valuable adjunct for determining the appropriate endpoint of coil embolization, especially in zone 2 TEVAR cases in which the distance between the left common carotid artery and LSA is short.

## Conclusions

NOGA serves as a valuable adjunct to TEVAR for optimizing stent graft coverage and confirming LSA embolization endpoints, potentially improving zone 2 repair outcomes.

## Funding

None.

## Disclosures

None.

## Appendix (online only)

Supplementary Video 1 NOGA visualization after the left subclavian artery coil embolization. NOGA imaging after left subclavian artery coil embolization demonstrates residual antegrade blood flow through small gaps between the coils, indicating incomplete embolization.

Supplementary Video 2 NOGA confirmation after additional coil placement. NOGA imaging after placement of an additional coil shows complete cessation of blood flow, confirming complete embolization.

## References

[1] Takahashi S., Komatsu S., Ohara T. (2018). Detecting intimal tear and subintimal blood flow of thrombosed acute aortic dissection with ulcer-like projections using non-obstructive angioscopy. J Cardiol Cases.

[2] Nishi H., Higuchi Y., Takahashi T. (2020). Aortic angioscopy assisted thoracic endovascular repair for chronic type B aortic dissection. J Cardiol.

[3] Czerny M., Schmidli J., Adler S. (2019). Current options and recommendations for the treatment of thoracic aortic pathologies involving the aortic arch: an expert consensus document of the European Association for Cardio-Thoracic Surgery (EACTS) & the European Society for Vascular Surgery (ESVS). Eur J Vasc Endovasc Surg.

[4] MacGillivray T.E., Gleason T.G., Patel H.J. (2022). The Society of Thoracic Surgeons/American Association for Thoracic Surgery clinical practice guidelines on the management of type B aortic dissection. J Thorac Cardiovasc Surg.

[5] Komatsu S., Ohara T., Takahashi S. (2015). Early detection of vulnerable atherosclerotic plaque for risk reduction of acute aortic rupture and thromboemboli and atheroemboli using non-obstructive angioscopy. Circ J.

[6] Sakakibara S., Nishi H., Kitahara M., Goto T., Nakazato T. (2023). Successful “PETTICOAT” procedure assisted by aortic angioscopy for complicated type B aortic dissection: case report. Int J Surg Case Rep.

[7] Parmer S.S., Carpenter J.P., Stavropoulos S.W. (2006). Endoleaks after endovascular repair of thoracic aortic aneurysms. J Vasc Surg.

[8] Azevedo A.I., Braga P., Rodrigues A. (2016). Persistent type I endoleak after endovascular treatment with chimney technique. Front Cardiovasc Med.

[9] Chaudhuri A., Tibballs J., Nadkarni S., Garbowski M. (2007). Digital embolization due to partially uncovered left subclavian artery post TEVAR: management with amplatzer vascular plug occlusion. J Endovasc Ther.

[10] Koganemaru M., Tanaka N., Nagata S., Abe T. (2017). Internal coil packing for mesh occlusion device. J Vasc Interv Radiol.

[11] Katada Y., Onozawa S., Takahashi S., Suzuki S. (2018). Ultrashort-segment embolization of high-flow vessels using a coil packing technique in an Amplatzer vascular plug. J Endovasc Ther.

[12] Maruhashi T., Nishimaki H., Ogawa Y., Chiba K., Kotoku A., Miyairi T. (2021). Preloading coil in plug technique for internal iliac artery embolization during endovascular abdominal aortic aneurysm repair. Cardiovasc Interv Radiol.

[13] Yasumoto T., Yakushiji H., Ohira R., Ochi S., Nakata S., Hirabuki N. (2015). Superselective coaxial microballoon-occluded coil emblization for vascular disorders: a preliminary report. J Vasc Interv Radiol.

